# Performance of Lung-RADS in different target populations: a systematic review and meta-analysis

**DOI:** 10.1007/s00330-023-10049-9

**Published:** 2023-08-30

**Authors:** Yifei Mao, Jiali Cai, Marjolein A. Heuvelmans, Rozemarijn Vliegenthart, Harry J. M. Groen, Matthijs Oudkerk, Marleen Vonder, Monique D. Dorrius, Geertruida H. de Bock

**Affiliations:** 1 Department of Epidemiology, University Medical Center Groningen, University of Groningen, 9700 RB Groningen, the Netherlands; 2 Department of Radiology, University Medical Center Groningen, University of Groningen, 9700 RB Groningen, the Netherlands; 3 Department of Pulmonary Diseases, University Medical Center Groningen, University of Groningen, 9700 RB Groningen, the Netherlands; 4 Institute for Diagnostic Accuracy, Prof. Wiersmastraat 5, 9713 GH Groningen, the Netherlands

**Keywords:** Lung neoplasms, Cancer screening, Solitary pulmonary nodule, X-ray computed tomography

## Abstract

**Objectives:**

Multiple lung cancer screening studies reported the performance of Lung CT Screening Reporting and Data System (Lung-RADS), but none systematically evaluated its performance across different populations. This systematic review and meta-analysis aimed to evaluate the performance of Lung-RADS (versions 1.0 and 1.1) for detecting lung cancer in different populations.

**Methods:**

We performed literature searches in PubMed, Web of Science, Cochrane Library, and Embase databases on October 21, 2022, for studies that evaluated the accuracy of Lung-RADS in lung cancer screening. A bivariate random-effects model was used to estimate pooled sensitivity and specificity, and heterogeneity was explored in stratified and meta-regression analyses.

**Results:**

A total of 31 studies with 104,224 participants were included. For version 1.0 (27 studies, 95,413 individuals), pooled sensitivity was 0.96 (95% confidence interval [CI]: 0.90–0.99) and pooled specificity was 0.90 (95% CI: 0.87–0.92). Studies in high-risk populations showed higher sensitivity (0.98 [95% CI: 0.92–0.99] vs. 0.84 [95% CI: 0.50–0.96]) and lower specificity (0.87 [95% CI: 0.85–0.88] vs. 0.95 (95% CI: 0.92–0.97]) than studies in general populations. Non-Asian studies tended toward higher sensitivity (0.97 [95% CI: 0.91–0.99] vs. 0.91 [95% CI: 0.67–0.98]) and lower specificity (0.88 [95% CI: 0.85–0.90] vs. 0.93 [95% CI: 0.88–0.96]) than Asian studies. For version 1.1 (4 studies, 8811 individuals), pooled sensitivity was 0.91 (95% CI: 0.83–0.96) and specificity was 0.81 (95% CI: 0.67–0.90).

**Conclusion:**

Among studies using Lung-RADS version 1.0, considerable heterogeneity in sensitivity and specificity was noted, explained by population type (high risk vs. general), population area (Asia vs. non-Asia), and cancer prevalence.

**Clinical relevance statement:**

Meta-regression of lung cancer screening studies using Lung-RADS version 1.0 showed considerable heterogeneity in sensitivity and specificity, explained by the different target populations, including high-risk versus general populations, Asian versus non-Asian populations, and populations with different lung cancer prevalence.

**Key Points:**

• *High-risk population studies showed higher sensitivity and lower specificity compared with studies performed in general populations by using Lung-RADS version 1.0.*

• *In non-Asian studies, the diagnostic performance of Lung-RADS version 1.0 tended to be better than in Asian studies.*

• *There are limited studies on the performance of Lung-RADS version 1.1, and evidence is lacking for Asian populations.*

**Supplementary information:**

The online version contains supplementary material available at 10.1007/s00330-023-10049-9.

## Introduction

Lung cancer remains the leading cause of cancer-related death, with a 5-year survival of just 10–20% [1]. Results from large-scale multicenter studies offer hope, showing that screening by low-dose computed tomography (LDCT) can detect lung cancer at an early stage [2–4]. Apart from structural differences in screening approaches, false-positive rates can be decreased without substantially decreasing the sensitivity of cancer detection by optimizing the definition of a positive screen [5, 6], and cost-effectiveness can be improved by ensuring appropriate management algorithms for positive results [7].

Protocols to perform lung cancer screening have been developed in Western countries. The American College of Radiology released “Lung CT Screening Reporting and Data System (Lung-RADS)” version 1.0 in 2014 [8] based on published caption data from the United States National Lung Screening Trial (NLST), Dutch–Belgian Lung cancer screening trial (NELSON), and International Early Lung Cancer Action Program (I-ELCAP) [2, 3, 9, 10]. Lung-RADS has become one of the most widely used reporting and management aids for screen-detected nodules worldwide. Applied to the NLST population, version 1.0 effectively and considerably decreased the false-positive rate, at the cost of only a slight decrease in the false-negative rate, by increasing the size threshold for a positive baseline screen from 4 mm (greatest diameter) to 6 mm (average diameter) [11]. Whereafter, the Korean Lung Cancer Screening Project (K-LUCAS), the first Asian population-based, multicenter prospective lung cancer screening program, also adopted version 1.0 [12].

Lung-RADS version 1.1 was published in 2019 [8] and introduced three major changes based on rolling evidence. First, it increased the upper size limit for non-solid nodules in Lung-RADS category 3 from 20 to 30 mm based on evidence that these non-solid nodules follow an indolent course [13, 14] with little risk when continuing annual follow-up [15, 16]. Second, it down-classified perifissural nodules measuring < 10 mm to Lung-RADS category 2 to reduce false positives given that these are typically benign [17, 18]. Third, it added volumetric measurements to monitor the nodule growth rate and improve the ability to predict malignancy [19, 20]. This revision further decreased the false-positive rate for a subset of NLST participants with non-calcified nodules [21]. Lung-RADS version 2022 was recently released (November 2022), and thus far, no published studies have evaluated this newest version [22].

Several systematic reviews or meta-analyses assessed the performance of lung cancer screening by LDCT [23, 24], but the included studies adopted various definitions of positive screens and management algorithms. We found no prior systematic validation of the diagnostic performance of a standardized management protocol in LDCT lung cancer screening, and the performance across different target populations, such as high-risk versus general populations and Asian versus non-Asian populations. This systematic review and meta-analysis aimed to evaluate the diagnostic performance of Lung-RADS (versions 1.0 and 1.1) for detecting lung cancer in different target populations and explore which characteristics of target population can impact the performance.

## Materials and methods

### Study design

We followed the Preferred Reporting Items for Systematic Reviews of Diagnostic Test Accuracy (PRISMA-DTA) guidelines [25] and the Cochrane Handbook for Systematic Reviews of Diagnostic Test Accuracy (version 2.0, 2022) [26], and registered our study protocol in the international prospective register of systematic reviews, or PROSPERO (no. CRD42022311028). Two radiologists (3 and 5 years’ experience in lung cancer screening) independently screened the literature, selected studies, collected and extracted data, and assessed quality, resolving differences of opinion by consensus or discussion with a third radiologist (> 10 years’ experience in lung cancer screening).

### Search strategy and literature screening

Literature searches of PubMed, Web of Science, Cochrane Library, and Embase were performed on 21 October 2022 using the strategies listed in Table 1, without language restrictions. Our search was based on the following keywords for lung cancer and Lung-RADS: (Lung Neoplasms OR lung OR pulmonary) AND (lung-RADS OR lungRADS OR lu-rads OR lurads OR RADS OR (reporting AND data-system)). And no filters were applied. Studies published before 2014 and duplicates were excluded using EndNote X8.

**Table 1:** Search strategy in PubMed, Embase, Cochrane, and Web of Science

Database	Search strategy
PubMed	(“Lung Neoplasms”[Mesh] OR lung[tiab] OR pulmonary[tiab]) AND (lung-RADS*[tiab] OR lungRADS*[tiab] OR lu-rads*[tiab] OR lurads*[tiab] OR RADS*[tiab] OR (reporting[tiab] AND data-system*[tiab]))
Embase	(“lung nodule”/exp OR “lung cancer”/exp OR “lung”/de OR (pulmonary OR lung):ti,ab,kw) AND (“lung imaging reporting and data system”/exp OR “reporting and data system”/de OR “data system”/de OR ((report* AND “data system*”) OR “lung rads*” OR lungrads* OR RADS):ti,ab,kw)
Cochrane	([mh “Lung Neoplasms”] OR lung:ti,ab OR pulmonary:ti,ab) AND (lung-RADS*:ti,ab OR lu-rads*:ti,ab OR lungRADS*:ti,ab OR RADS:ti,ab OR (reporting:ti,ab NEAR/5 data-system*:ti,ab))
Web of Science	TS = (lung OR pulmonary) AND TS = ((report* AND “data system*”) OR “lung rads*” OR lungrads* OR “lu rads*” OR lurads* OR RADS)

We screened titles, abstracts, and full texts of articles, and selected studies containing data for populations (a) screened for lung cancer, (b) screened by LDCT, (c) where a Lung-RADS protocol was applied for lung nodule management, (d) where the clinical or histopathological diagnosis of lung cancer was used as the reference standard, and (e) that included the diagnostic performance of Lung-RADS. Studies were excluded if they met any of the following criteria: (a) review, case reports, conference abstracts, editorials, or book chapters; (b) studies without sufficient data on 2 × 2 contingency tables; and (c) studies with data from the same cohort. When articles used data from the same cohort, we only included the article that included the largest population.

### Data extraction and definitions

We used standardized data extraction forms to collect the following items from each included study: first author name, publication year, country, Lung-RADS version, study design (retrospective or prospective), baseline inclusion period, number of screened participants, number of positive screens, number of lung cancers, age (mean, median, and range), sex, smoking status, lung nodule number and type (solid, part solid, or ground glass), reference standard for lung cancer, and eligibility criteria. The reference standard for lung cancer was grouped as “pathology alone” (e.g., pathological proof only) or “pathology and other methods” (e.g., pathological proof or repeat CT, PET, multidisciplinary consensus). If some data were not available in the main text, supplementary files, or their references, we contacted the authors to resolve the missing data.

The Lung-RADS algorithm distinguishes baseline and follow-up screenings, with nodule categorization based on nodule type and size at baseline, and then also considering nodule pre-existence and growth rate at follow-up. Each Lung-RADS category has specific management recommendations: categories 1 and 2 indicate “negative screens” suitable for continued annual screening, while categories 3 and 4 indicate “positive screens” suitable for referral for additional screening or interventions before the next annual screening [8].

Finally, we recorded the population type, population area, and lung cancer prevalence from the extracted data, and studies were stratified based on these items. First, for population type, this was stratified into “high risk” and “general” based on smoking status. Studies with high-risk populations either used inclusion criteria based on smoking status (e.g., NLST selection criteria [2], NCCN high-risk criteria [27], USPSTF criteria [28], PLCOm2012 [29]) or only included participants who smoked. By contrast, studies with general populations either used inclusion criteria based on factors other than the smoking history or included both current, former, and never smokers. Second, studies were stratified by their geographic area into Asia and non-Asia groups. Third, regarding the prevalence of lung cancer, studies were stratified into two groups based on the median prevalence [30].

### Quality assessment

The quality of included studies was evaluated with the Quality Assessment of Diagnostic Accuracy Studies (QUADAS-2) tool (Table S1) in Review Manager (RevMan, version 5.4. Copenhagen: The Cochrane Collaboration, 2020). QUADAS-2 is a structured checklist comprising four domains, namely patient selection, index test, reference standard, and flow and timing. The risk of bias was assessed using two or three signaling questions for each of the four domains, and concern about applicability was evaluated with one signaling question for the first three domains only. These seven items were then judged as “low,” “high,” or “unclear” [31].

### Data synthesis and analysis

To calculate the sensitivity and specificity for lung cancer detection in the included studies, we constructed 2 × 2 contingency tables. A bivariate random-effects model was used to estimate the pooled sensitivity and specificity with their 95% CIs and represented in forest plots. Heterogeneity among studies was assessed using the Cochran *Q* test (*p* < 0.05 indicated heterogeneity) and Higgins inconsistency index (*I*^2^ ≥ 50% suggested substantial heterogeneity) [32, 33]. If heterogeneity was observed, we performed stratified and meta-regression analyses to explore the likely source. Stratified analyses were performed based on the following covariates: population type (high risk or general population), population area (Asia or non-Asia), study design (retrospective or prospective), reference standard (pathology alone, or pathology and other methods), and lung cancer prevalence (less or more than median). Publication bias was tested using Deeks’ funnel plot. For data analysis, the Midas module [34] in Stata 15.0 (StataCorp) was applied in the present study.

## Results

### Study selection and characteristics

**Fig. 1:**
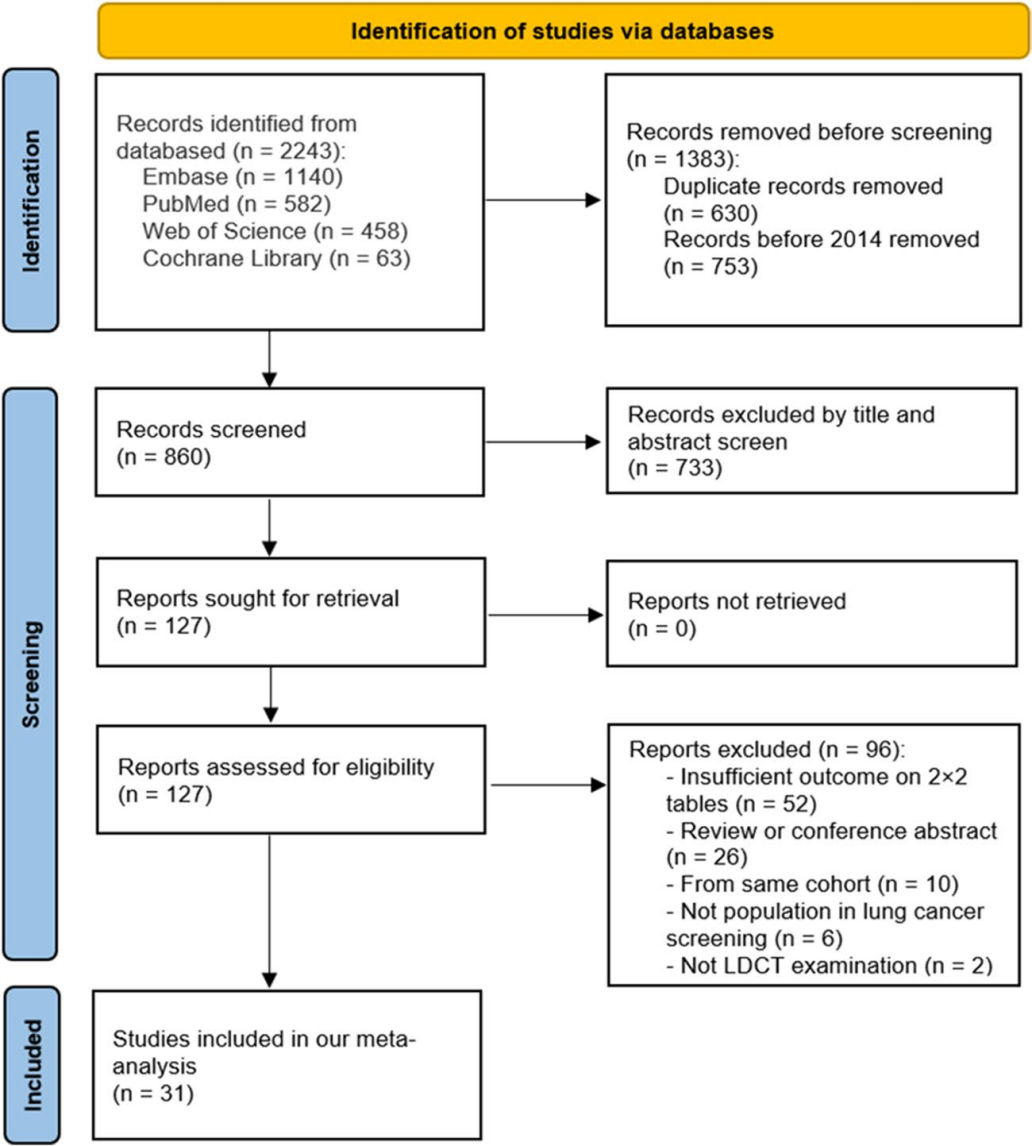
PRISMA 2020 flow diagram for the literature search and screening

Figure 1 shows the literature search and study selection processes. The initial search yielded 2243 articles, from which we excluded 753 studies published before 2014 and a further 630 duplicates. We then excluded 733 articles by title and abstract screening and a further 96 by full-text screening. This left 31 eligible articles [11, 21, 35–63] comprising 104,224 participants for analysis.

Table 2 shows the study and patient characteristics for the included articles. Of the 31 studies, 27 only evaluated version 1.0, three only evaluated version 1.1, and one assessed both versions, but only the version 1.1 result was extracted. All included studies applied Lung-RADS at baseline screening, and only two (11, 57) separately evaluated it at baseline and follow-up. The studies were performed in nine countries, with the USA (54.8%, 17/31), China (9.7%, 3/31), South Korea (9.7%, 3/31), and Canada (6.5%, 2/31) as the most common. For the high-risk population, we identified 24 studies [11, 21, 35, 37–39, 43–46, 48–50, 52–62] that included only current or former smokers and one study that included 99.3% current or former smokers [36]. For the general population, we only identified five studies [40, 41, 47, 51, 63] that included both current, former, and never smokers and one study [42] that included only female never smokers. The median prevalence of lung cancer was 1.9% (range, 0.5–7.8%) for version 1.0 studies, including 14 with ≥ 1.9% prevalence, and 13 with < 1.9% prevalence, and was 2.4% (range, 2.0–3.9%) for version 1.1 studies.

**Table 2:** Characteristics of included studies and participants

Author, year [reference]	Country	Lung-RADS version	Study design	Inclusion period	No. of included patients	Age (y)	Sex (male)	Smoking status	Lung nodule (type and no.)	Reference standard	Eligibility criteria	Lung cancer prevalence
Pinsky 2015 [11]	USA	1.0	Retrospective cohort	2002–2004	26,455	Range 55–74	59%	48.1% current and 51.9% former smokers	All types; unknown number	Histopathology	NLST selection criteria^*^	1.1% (292)
McKee 2015 [35]	USA	1.0	Retrospective cohort	January 2012–May 2014	1603	64	53%	45.8% current and 54.2% former smokers	All types; unknown number	Histopathology, PET, suspicious growth rate, or multidisciplinary consensus	NCCN high-risk criteria^#^	1.8% (29)
Halpenny 2016 [36]	USA	1.0	Retrospective cohort	May 2011–September 2014	139	66	43%	99.3% current or former smokers	57.1% (4) malignant SN, 28.6% (2) malignant PSN, and 14.3% (1) malignant GGN	Histopathology	A previous history of malignancy	5.0% (7)
Guichet 2017 [37]	USA	1.0	Prospective cohort	July 2015–April 2017	275	59	52%	81.1% current and 18.9% former smokers	All types; unknown number	Histopathology, FU	NCCN high-risk criteria^#^	0.7% (2)
Jacobs 2017 [38]	USA	1.0	Prospective cohort	June 2014–December 2015	680	64	55%	48.3% current and 51.7% former smokers	All types; unknown number	Histopathology	Age 55–77, ≥ 30 pack-year smoking, quit < 15 years	2.4% (16)
Marshall 2017 [39]	Australia	1.0	Retrospective cohort	December 2007–December 2010	256	65	67%	47.3% current and 52.7% former smokers	All types; 136 patients with 301 nodules	Histopathology	NLST selection criteria^*^	2.0% (5)
Hsu 2018 [40]	China, Taiwan	1.0	Retrospective cohort	August 2013–October 2014	1978	57	55%	10.7% current, 16.5% former, and 72.8% never smokers	All types; unknown number	Histopathology	Age 40–80 years	1.6% (32)
Kang 2018 [41]	South Korea	1.0	Retrospective cohort	March 2003–June 2016	28,807	52	71%	57.8% current or former smokers, and 42.2% never smokers	1218 never smokers with nodules: 34.3% (418) with SN, 10.1% (123) with PSN, and 55.5% (677) with GGN; unknown no. for smokers	Histopathology	No special	0.7% (198)
Kim 2018 [42]	South Korea	1.0	Retrospective cohort	August 2002–December 2007	4365	51	0%	100% never smokers	18.2% (4) malignant SN, 50% (11) malignant PSN, 18.2% (4) malignant GGN	Histopathology	Age 40–79 years, never smokers, female	0.5% (22)
Bhandaria 2019 [43]	USA	1.0	Retrospective cohort	2016–2017	4500	62	46%	69.0% current and 31.0% former smokers	All types; unknown number	Histopathology	USPSTF criteria^&^	1.6% (70)
Healey 2019 [44]	USA	1.0	Prospective cohort	January 2015–January 2017	952	NA	NA	100% current or former smokers	All types; unknown number	Histopathology	NLST selection criteria^*^	1.9% (18)
Kaminetzky 2019 [45]	USA	1.0	Prospective cohort	2012–2016	1181	64	48%	71.4% current and 28.6% former smokers	All types; unknown number	Histopathology, or imaging, National Death Index	NLST selection criteria^*^	2.5% (30)
Meier-Schroers 2019 [46]	Germany	1.0	Prospective cohort	NA	224	59	NA	100% current or former smokers	All types; unknown number	Histopathology	Age 50–70, ≥ 15 cigarettes/day for ≥ 25 or ≥ 10 cigarettes/day for ≥ 30 years, current or former quit < 10 years	3.6% (8)
Ouyang 2019 [47]	China	1.0	Prospective cohort	2018	1372	59	37%	NA	69.6% (955) participants without nodules, 25.0% (343) with SN, 4.2% (57) with PSN, and 1.2% (17) with GGN	Histopathology	Age ≥ 45 years	0.7% (9)
Teles 2019 [48]	Brazil	1.0	Prospective cohort	May 2015–April 2016	472	51	76%	60.0% current and 40% former smokers	All types; unknown number	Histopathology	Age > 45, current or former smoker	0.6% (3)
Tremblay 2019 [49]	Canada	1.0	Retrospective cohort	June 2015–December 2017	775	63	50%	45.2% current and 54.8% former smokers	All types; unknown number	Histopathology	NLST criteria^*^, or age 55–80 with estimated 6-year risk (PLCO_m2012_) ≥ 1.5%	2.8% (22)
Barbosa 2020 [50]	USA	1.0	Retrospective cohort	May 2014–July 2019	260	65.5	48%	55.0% current and 45.0% former smokers	33% participants without nodules, 62% with SN, 1% with PSN, and 4% with GGN	Histopathology	Age 55–80, ≥ 30 pack-year smoking, quit < 15 years	1.5% (4)
Hsu 2020 [51]	China, Taiwan	1.0	Prospective cohort	February 2017–August 2018	836	NA	43%	NA	All types; unknown number	Histopathology	Age 40–80	3.2% (27)
Kessler 2020 [52]	USA	1.0	Prospective cohort	December 2012–June 2016	486	63	46%	66.7% current and 33.3% former smokers	All types; unknown number	Histopathology	NLST selection criteria^*^	7.8% (38)
Kim 2020 [53]	South Korea	1.0	Prospective cohort	April 2017–December 2018	11,394	62	97%	53.3% current and 46.7% former smokers	All types; unknown number	Histopathology	NLST selection criteria^*^	0.6% (65)
Maller 2020 [54]	USA	1.1	Retrospective cohort	2011–2019	454	65	49%	29.5% current and 61.5% former smokers	All types; unknown number	Histopathology	NCCN high-risk criteria^#^	2.4% (11)
O’Dwyer 2020 [55]	USA	1.0	Prospective cohort	May 2011–November 2018	543	66	43%	100% current or former smokers	74.3% (26) malignant SN, 11.4% (4) malignant PSN, and 14.3% (5) malignant GGN	Histopathology	NCCN high-risk criteria^#^, and a previous history of malignancy	6.4% (35)
White 2020 [56]	USA	1.0	Retrospective cohort	September 2013–September 2018	962	66	70%	100% current or former smokers	All types; unknown number	Histopathology or FU	USPSTF criteria^&^	4.4% (42)
Darling 2021 [57]	Canada	1.0	Prospective cohort	June 2017–May 2018	1624	NA	51%	58.9% current and 41.1% former smokers	All types; unknown number	Histopathology	6-year risk (PLCO_m2012_) ≥ 2%	1.7% (28)
Erkmen 2021 [58]	USA	1.0	Prospective cohort	October 2015–March 2018	496	64	50%	61.0% current and 39.0% former smokers	All types; unknown number	Histopathology or repeat CT	Age 55–77, ≥ 30 pack-year smoking, quit < 15 years	3.2% (16)
Kastner 2021^ǂ^ [21]	USA	1.0, and 1.1	Retrospective cohort	August 2002–April 2004	2813	62	61%	100% current or former smokers	Only non-calcified nodules: 92.5% (4078) SN, 1.9% (82) PSN, and 5.6% (248) GGN	Histopathology	NLST selection criteria^*^	3.9% (110)
Parang 2021 [59]	India	1.0	Retrospective cohort	January 2016–January 2019	350	61	96%	100% current or former smokers	All types; unknown number	Histopathology	Smoker, current or former quit < 15 years	2.0% (7)
Regis 2021 [60]	USA	1.1	Retrospective cohort	January 2012–December 2018	4301	63.1	56%	47.4% current and 52.6% former smokers	All types; unknown number	Histopathology, nodule growth, PET/CT, or multidisciplinary consensus	NCCN eligibility criteria^#^	2.0% (85)
Silva 2021 [61]	Italy	1.1	Retrospective cohort	NA	1248	Range 55–74	72%	66.3% current and 33.7% former smokers	57.3% (715) participants without nodules, 31.0% (387) with SN, 2.4% (30) with PSN, and 9.3% (116) with GGN	Histopathology	NLST selection criteria^*^	2.6% (32)
Oshiro 2022 [62]	USA	1.0	Retrospective cohort	January 2015–December 2019	838	65.5	61%	100% current or former smokers	All types; unknown number	Histopathology	Age 55–79, ≥ 30 pack-year smoking, quit < 15 years	1.6% (13)
Panina 2022 [63]	Kazakhstan	1.0	Prospective cohort	June 2018–May 2020	3671	Range 40–75	43%	26.1% current, 14.2% former, and 59.9% never smokers	83.8% (62) malignant SN, and 16.2% (12) malignant PSN	Histopathology	Age 40–75	2.0% (74)

*NLST* National Lung Screening Trial; *NCCN* National Comprehensive Cancer Network; *USPSTF* US Preventive Services Task Force; *PLCOm2012* risk prediction models including Tammemägi’s modified Prostate, Colorectal, Lung, and Ovarian Cancer Screening Trial 2012; *NA* not applicable; *FU* follow-up; *SN* solid nodule; *PSN* part-solid nodule; *GGN* ground-glass nodule

^*^NLST selection criteria: age 55–74, 30 pack-ear smoking history, quit within 15 years

^#^NCCN high-risk criteria: (1) age 55–74 years, ≥ 30 pack-year smoking history, quit within 15 years; (2) age > 50 years, ≥ 30 pack-year smoking history

^&^USPSTF criteria: age 55–80 years, ≥ 30 pack-year smoking history, quit within 15 years

^ǂ^The study by Kastner [21], which evaluated both versions 1.0 and 1.1, used the same database (NLST data) as the one by Pinsky [11], which only evaluated version 1.0, but with a smaller population. Therefore, we only extracted the result of the diagnostic performance of Lung-RADS version 1.1 from the study by Kastner

### Study quality

Overall, 13 studies satisfied all seven items of the QUADAS-2 checklist (Figure S1 and Table S2), 28 satisfied at least six items, and all 31 satisfied at least four items, suggesting a reasonable overall study quality (Figure S1). In the patient selection domain, two studies [21, 52] showed high risk of bias and high concern for applicability; these studies only included patients with non-calcified nodules, thereby excluding Lung-RADS category 1 (patients with calcified nodules and without nodules). Regarding flow and timing, nine studies showed an unclear risk of bias and six showed a high risk of bias. Eight studies [36, 43, 44, 46–48, 50, 55, 59] showed unclear risk of bias because they lacked sufficient information to determine the interval between Lung-RADS classification and lung cancer diagnosis. Among the studies with a high risk of bias, two [21, 35] included less than 90% of screened participants in the evaluation of Lung-RADS and six [35, 36, 45, 56, 60] applied different reference standards for lung cancer diagnosis. All included studies provided optimal scores for the index and reference standards.

### Diagnostic performance of Lung-RADS version 1.0

**Fig. 2:**
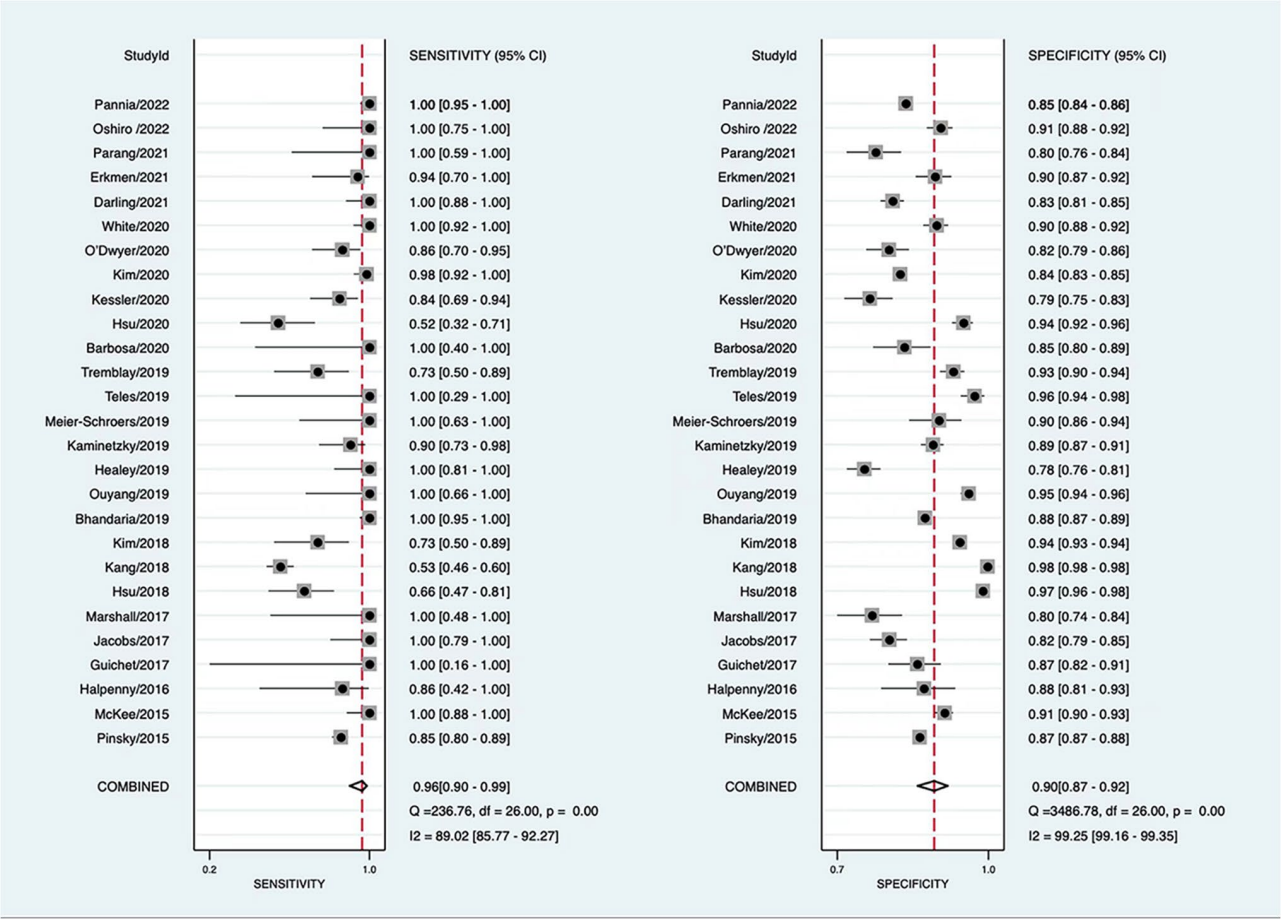
Forest plot for the pooled sensitivity (left) and specificity (right) of Lung-RADS version 1.0

The diagnostic performance of Lung-RADS version 1.0 at baseline was assessed in 27 studies with 95,413 participants [11, 35–53, 55–59, 62, 63]. These showed a pooled sensitivity of 0.96 (95% CI: 0.90–0.99) and a pooled specificity of 0.90 (95% CI: 0.87–0.92) (Fig. 2 and Table 3). We found substantial heterogeneity for both sensitivity (*p* < 0.001, *I*^2^ = 89.0%) and specificity (*p* < 0.001, *I*^2^ = 99.3%) across the studies. Meta-regression analysis showed that population type (*p* < 0.001), population area (*p* = 0.02), and lung cancer prevalence (*p* = 0.02) were significant covariates affecting heterogeneity among studies using version 1.0 (Table 3). Neither study design nor reference standard for lung cancer posed significant sources of heterogeneity (*p* > 0.05). Overall, the high-risk populations (*n* = 21; 54,384 individuals) showed higher sensitivity (0.98; 95% CI: 0.92–0.99) and lower specificity (0.87; 95% CI: 0.85–0.88) compared with general populations (*n* = 6; 41,029 individuals), where the corresponding values were 0.84 (95% CI: 0.50–0.96) and 0.95 (95% CI: 0.92–0.97), respectively (Table 3). The 95% CIs for both sensitivity and specificity overlapped when comparing Asia and non-Asia areas. However, studies outside Asia (*n* = 19; 49,102 individuals) tended to have a higher sensitivity (0.97; 95% CI: 0.91–0.99) and lower specificity (0.88; 95% CI: 0.85–0.90) compared with those performed in Asia (*n* = 8, 46,311 individuals), where the sensitivity was 0.91 (95% CI: 0.67–0.98) and the specificity was 0.93 (95% CI: 0.88–0.96) (Table 3). In addition, 95% CIs overlapped when comparing groups by a lung cancer prevalence < 1.9% (*n* = 13; 83,870 individuals; sensitivity, 0.97 [0.84–0.99]; specificity, 0.92 [0.88–0.94]) and ≥ 1.9% (*n* = 14; 11,543 individuals; sensitivity, 0.96 [0.86–0.99]; specificity, 0.87 [0.84–0.89]) (Table 3).

**Table 3:** Overall diagnostic performance of each subset and the stratified or meta-regression analysis result for version 1.0

Parameter	Sensitivity	Specificity	Meta-regression analysis (*p* value)
*Lung-RADS version*			0.03
Version 1.0 subset (*n* = 27)	0.96 [0.90–0.99]	0.90 [0.87–0.92]	
Version 1.1 subset (*n* = 4)	0.91 [0.83–0.96]	0.81 [0.67–0.90]	
Version 1.0 subset			
*Population type*			< 0.001
High-risk population (*n* = 21)	0.98 [0.92–0.99]	0.87 [0.85–0.89]	
General population (*n* = 6)	0.84 [0.50–0.96]	0.95 [0.92–0.97]	
*Population area*			0.02
Non-Asia (*n* = 19)	0.97 [0.91–0.99]	0.88 [0.85–0.90]	
Asia (*n* = 8)	0.91 [0.67–0.98]	0.93 [0.88–0.96]	
*Study design*			0.45
Prospective (*n* = 14)	0.97 [0.88–0.99]	0.88 [0.84–0.91]	
Retrospective (*n* = 13)	0.96 [0.82–0.99]	0.91 [0.87–0.94]	
*Reference standard*			0.22
Pathology alone (*n* = 21)	0.94 [0.84–0.98]	0.90 [0.86–0.92]	
Pathology and other methods (*n* = 6)	0.99 [0.88–1.00]	0.89 [0.88–0.90]	
*Prevalence of lung cancer*			0.02
< 1.9% (*n* = 13)	0.93 [0.83–0.98]	0.92 [0.88–0.94]	
≥ 1.9% (*n* = 14)	0.96 [0.86–0.99]	0.87 [0.84–0.89]	

The median lung cancer prevalence was 1.9% among the included studies

### Diagnostic performance of Lung-RADS version 1.1

**Fig. 3:**
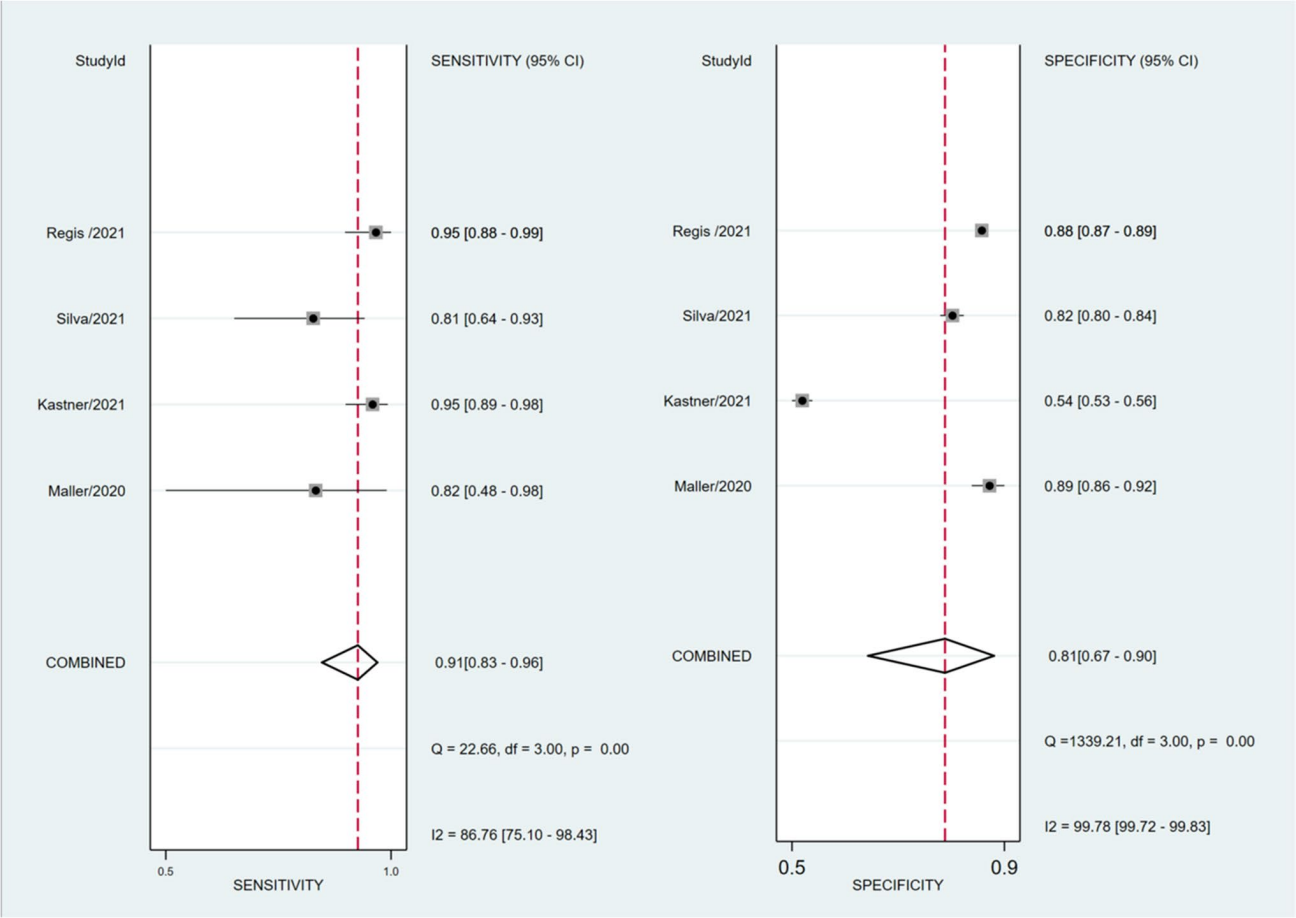
Forest plot for the pooled sensitivity (left) and specificity (right) of Lung-RADS version 1.1

Four studies with 8811 participants [21, 54, 60, 61], all comprising high-risk populations outside Asia, examined the diagnostic performance of Lung-RADS version 1.1 at baseline. The pooled sensitivity and specificity were 0.91 (95% CI: 0.83–0.96) and 0.81 (95% CI: 0.67–0.90), respectively (Fig. 3 and Table 3). We found substantial heterogeneity for both sensitivity (*p* < 0.001, *I*^2^ = 86.8%) and specificity (*p* < 0.001, *I*^2^ = 99.5%) among the studies.

### Publication bias

Studies that assessed Lung-RADS version 1.0 showed no publication bias according to Deeks’ funnel plot (*p* = 0.44; Figure S2). However, publication bias could not be analyzed for Lung-RADS version 1.1 because of the limited number of studies.

## Discussion

In this systematic review and meta-analysis, we assessed the diagnostic performances of Lung-RADS in lung cancer screening by LDCT. Our data showed that Lung-RADS version 1.0 had a pooled sensitivity of 96% (95% CI: 0.90–0.99) and specificity of 90% (95% CI: 0.87–0.92). Lung-RADS version 1.1 retained a similar pooled sensitivity (0.91; 95% CI: 0.83–0.96) but had suboptimal specificity (0.81; 95% CI: 0.67–0.90), which was based on a limited number of non-Asian studies.

For version 1.0, our results showed that studies in high-risk populations had higher sensitivity and somewhat lower specificity than studies in general populations. This suggests that Lung-RADS version 1.0 is more applicable to high-risk populations, consistent with the development of version 1.0 with published data from lung cancer screening trials that targeted high-risk populations [2, 3, 9, 10]. In addition, studies of version 1.0 outside Asia tended to show higher sensitivity and lower specificity compared with studies in Asia. Notably, all 19 studies outside Asia targeted high-risk populations, contrasting with only two out of eight in Asia [53, 59]. Nonetheless, compared with the studies outside Asia, both studies in high-risk Asian populations showed similar sensitivities and lower specificities. Studies in general Asian populations (*n* = 6) showed lower sensitivities and higher specificities than those studies, but due to the limited number in high-risk populations, we could not draw firm conclusions about either the most applicable population type for version 1.0 in Asia or where version 1.0 could have the greatest effect for those at high risk.

Lung-RADS was designed for the lung cancer screening, and as such, this meta-analysis only included screening studies. Most screening studies included high-risk populations, and in these studies, a high-risk population is mainly determined by smoking criteria. However, evidence has now shown that the proportion of lung cancer is higher in Asian than in Western never smokers, with about one-third of lung cancers in East Asia found in never smokers [64, 65]. Therefore, it may be reasonable that in the future, eligibility criteria for lung cancer screening will be extended, and screening will also be performed in never or less heavy smokers [66]. Nonetheless, our meta-analysis also indicates that targeting a general population using Lung-RADS version 1.0 in Asia will not necessarily result in the same high sensitivity found outside Asia in high-risk populations. This underscores the fact that the definition for “high risk” needs to be optimized and that the classification in Lung-RADS might need revising to improve its applicability in Asia. Other risk factors, such as emphysema, passive smoking, air pollution, fireplace fume exposure, and family history of lung cancer, may be included in the eligibility criteria [66, 67]. Moreover, in the latest 2021 USPSTF Criteria, the minimum age of eligibility criteria was lowered from 55 to 50 years, and other scientific societies may consider lowering the starting age as well [28].

Lung-RADS version 1.1 tended to show lower sensitivity and specificity compared with version 1.0, although the 95% CIs for both overlapped. We only identified four studies using Lung-RADS version 1.1. Among them, although the study by Kastner et al [21] evaluated both versions of Lung-RADS, we only extracted their results for version 1.1. Moreover, the study included participants with non-calcified nodules and excluded those with Lung-RADS category 1. This have undoubtedly led to an underestimation in the specificity of version 1.1 compared with the other studies. Thus, we cannot reach a reliable conclusion about the diagnostic performance of Lung-RADS version 1.1, or indeed, determine which version performs better for LDCT lung cancer screening in different areas. By contrast, Kastner et al [21] have reported conflicting results; Lung-RADS version 1.1 outperformed version 1.0, with the more recent version showing higher specificity at the cost of only a minimal decrease in sensitivity. It should be noted that their study population was at high risk (NLST population), whereas the current meta-analysis also included studies in general populations. Moreover, all four studies using version 1.1 were performed outside Asia. Therefore, more studies are needed to give more precise estimates of the diagnostic performance of Lung-RADS version 1.1 for lung cancer screening, especially in Asia.

Only one recently published meta-analysis on the performance of LDCT lung cancer screening reported the pooled sensitivity and specificity of Lung-RADS protocol in the stratified analysis, but without further discussion [24]. This recent meta-analysis included only nine studies using Lung-RADS version 1.0 and no studies using version 1.1. Additionally, only one study was performed in an Asian general population. Thus, our current study expands the evidence to Lung-RADS version 1.1 and Asian population.

Considerable heterogeneity was noted among studies using both versions. Stratified and meta-regression analyses revealed that population type, population area, and lung cancer prevalence could explain part of heterogeneity among studies using Lung-RADS version 1.0. Generally, population type and area affect the population case mix (e.g., participants requiring additional tests or procedures) and disease prevalence, which could have caused the diagnostic accuracy of Lung-RADS to vary [68, 69]. Thus, these three factors are expected causes of study heterogeneity when using Lung-RADS.

This study has several limitations. First, we could not evaluate Lung-RADS management algorithms for follow-up screening, due to the limited number of studies that evaluated Lung-RADS at follow-up. Second, the proportion of lung cancers manifesting as subsolid nodules is much higher in Asia than in Western countries [70], which could result in the differences of Lung-RADS performance between Asia and non-Asia. In addition, to decrease the false-positive rates, version 1.1 added the identification and classification of perifissural nodules. However, only five included studies reported the proportion of subsolid nodules [21, 41, 47, 50, 61], and only one mentioned that of perifissural nodules [21]. Due to limited data, we could not evaluate the performance of Lung-RADS by nodule type, e.g., subsolid or perifissural nodules. Third, the included studies contained inadequate data about follow-up duration for lung cancer diagnosis, the experience of radiologists who read CT scans, and the quality of the CT images, so we cannot assess the impact of these factors on heterogeneity. Fourth, we identified only a few studies using version 1.1, likely because of its comparatively recent publication in 2019. The study by Kastner et al that accounted for a large proportion of the version 1.1 subset also showed a high risk of selection bias, preventing any firm conclusion about which version had the better diagnostic performance.

In conclusion, this systematic review and meta-analysis showed that there is a considerable heterogeneity in sensitivity and specificity among lung cancer screening studies using Lung-RADS version 1.0, explained by population type (high-risk versus general), population area (Asia versus non-Asia), and lung cancer prevalence. There are limited studies using Lung-RADS version 1.1 and data is lacking for Asian populations.

### Supplementary Information

Below is the link to the electronic supplementary material.

Supplementary file1 (PDF 459 KB)

## Abbreviations

CI: Confidence interval
LDCT: Low-dose computed tomography
Lung-RADS: Lung CT Screening Reporting and Data System
NLST: National Lung Screening Trial
QUADAS: Quality Assessment of Diagnostic Accuracy Studies
