# Supplementary material for: Performance of Lung-RADS in different target populations: a systematic review and meta-analysis
Source: Eur Radiol. 2023 Aug 30;34(3):1877–92. doi: 10.1007/s00330-023-10049-9 (PMC10873443; doi:10.1007/s00330-023-10049-9)
Supplement: Supplementary file 1 — Supplementary file1 (PDF 459 KB) [file 330_2023_10049_MOESM1_ESM.pdf]

Performance of Lung-RADS in Different Target Populations: A Systematic Review and Meta-Analysis

Electronic Supplementary Material (ESM)

Eur Radiol (2023) Mao Y, Cai J, Heuvelmans MA et al.

Table S1. Quality Assessment of Diagnostic Accuracy Studies (QUADAS-2) tool, version 2.

Domain	Description	Rate
1. Patient selection		
Risk of Bias	Could the selection of patients have introduced bias?	Risk: Low/High/Unclear
Signaling questions	Was a consecutive or random sample of patients enrolled?	Yes/No/Unclear
	Was a case-control design avoided?	Yes/No/Unclear
	Did the study avoid inappropriate exclusions?	Yes/No/Unclear
Applicability	Are there concerns that the included patients and setting do not match the review question?	Risk: Low/High/Unclear
2. Index Test		
Risk of Bias	Could the conduct or interpretation of the index test have introduced bias?	Risk: Low/High/Unclear
Signaling questions	Were the index test results interpreted without knowledge of the results of the reference standard?	Yes/No/Unclear
	If a threshold was used, was it prespecified?	Yes/No/Unclear
Applicability	Are there concerns that the index test, its conduct, or interpretation differ from the review question?	Risk: Low/High/Unclear
3. Reference Standard		
Risk of Bias	Could the reference standard, its conduct, or its interpretation have introduced bias?	Risk: Low/High/Unclear
Signaling questions	Is the reference standard likely to correctly classify the target condition?	Yes/No/Unclear
	Were the reference standard results interpreted without knowledge of the results of the index test?	Yes/No/Unclear
Applicability	Are there concerns that the target condition as defined by the reference standard does not match the question?	Risk: Low/High/Unclear
4. Flow and Timing		
Risk of Bias	Could the patient flow have introduced bias?	Risk: Low/High/Unclear
Signaling questions	Was there an appropriate interval between index test (s) and reference standard?	Yes/No/Unclear
	Did all patients receive the same reference standard?	Yes/No/Unclear
	Were more than 90% of patients included in the analysis?	Yes/No/Unclear

Table S2. Details of quality assessment result.

Study	Risk of Bias														Applicability		
	Patient selection				Index Test			Reference Standard			Flow and Timing				Patient selection	Index Test	Reference Standard
	Q1	Q2	Q3	Risk	Q1	Q2	Risk	Q1	Q2	Risk	Q1	Q2	Q3	Risk	Risk	Risk	Risk
Pinsky 2015	Y	Y	Y	L	Y	Y	L	Y	Y	L	Y	Y	Y	L	L	L	L
McKee 2015	Y	Y	Y	L	Y	Y	L	Y	Y	L	Y	N	N	H	L	L	L
Halpenny 2016	Y	Y	Y	L	Y	Y	L	Y	Y	L	U	Y	Y	U	L	L	L
Guichet 2017	Y	Y	Y	L	Y	Y	L	Y	Y	L	Y	N	Y	H	L	L	L
Jacobs 2017	Y	Y	Y	L	Y	Y	L	Y	Y	L	Y	Y	Y	L	L	L	L
Marshall 2017	Y	Y	Y	L	Y	Y	L	Y	Y	L	Y	Y	Y	L	L	L	L
Hsu 2018	Y	Y	Y	L	Y	Y	L	Y	Y	L	Y	Y	Y	L	L	L	L
Kang 2018	Y	Y	Y	L	Y	Y	L	Y	Y	L	Y	Y	Y	L	L	L	L
Kim 2018	Y	Y	Y	L	Y	Y	L	Y	Y	L	Y	Y	Y	L	U	L	L
Bhandaria 2019	Y	Y	Y	L	Y	Y	L	Y	Y	L	U	Y	Y	U	L	L	L
Bihan 2019	Y	Y	Y	L	Y	Y	L	Y	Y	L	U	Y	Y	U	H	L	L
Healey 2019	Y	Y	Y	L	Y	Y	L	Y	Y	L	U	Y	Y	U	L	L	L
Kaminetzky 2019	Y	Y	Y	L	Y	Y	L	Y	Y	L	Y	N	Y	H	L	L	L
Meier-Schroers 2019	Y	Y	Y	L	Y	Y	L	Y	Y	L	U	Y	Y	U	L	L	L
Teles 2019	Y	Y	Y	L	Y	Y	L	Y	Y	L	U	Y	Y	U	L	L	L
Tremblay 2019	Y	Y	Y	L	Y	Y	L	Y	Y	L	Y	Y	Y	L	L	L	L
Barbosa 2020	Y	Y	Y	L	Y	Y	L	Y	Y	L	U	Y	Y	U	L	L	L
Hsu 2020	Y	Y	Y	L	Y	Y	L	Y	Y	L	Y	Y	Y	L	L	L	L
Kessler 2020	Y	Y	N	H	Y	Y	L	Y	Y	L	Y	Y	Y	L	H	L	L
Kim 2020	Y	Y	Y	L	Y	Y	L	Y	Y	L	Y	Y	Y	L	L	L	L
Maller 2020	Y	Y	Y	L	Y	Y	L	Y	Y	L	Y	Y	Y	L	L	L	L
O’Dwyer 2020	Y	Y	Y	L	Y	Y	L	Y	Y	L	U	Y	Y	U	L	L	L
White 2020	Y	Y	Y	L	Y	Y	L	Y	Y	L	U	N	Y	H	L	L	L
Darling 2021	Y	Y	Y	L	Y	Y	L	Y	Y	L	Y	Y	Y	L	L	L	L
Erkmen 2021	Y	Y	Y	L	Y	Y	L	Y	Y	L	Y	N	Y	H	L	L	L
Kastner 2021	Y	Y	N	H	Y	Y	L	Y	Y	L	Y	Y	N	H	H	L	L
Parang 2021	Y	Y	Y	L	Y	Y	L	Y	Y	L	U	Y	Y	U	L	L	L
Regis 2021	Y	Y	Y	L	Y	Y	L	Y	Y	L	Y	N	Y	H	L	L	L
Silva 2021	Y	Y	Y	L	Y	Y	L	Y	Y	L	Y	Y	Y	L	L	L	L
Oshiro 2022	Y	Y	Y	L	Y	Y	L	Y	Y	L	Y	Y	Y	L	L	L	L
Panina 2022	Y	Y	Y	L	Y	Y	L	Y	Y	L	Y	Y	Y	L	L	L	L

Note: Y=Yes, N=No, U=Unclear, L=Low, H=High

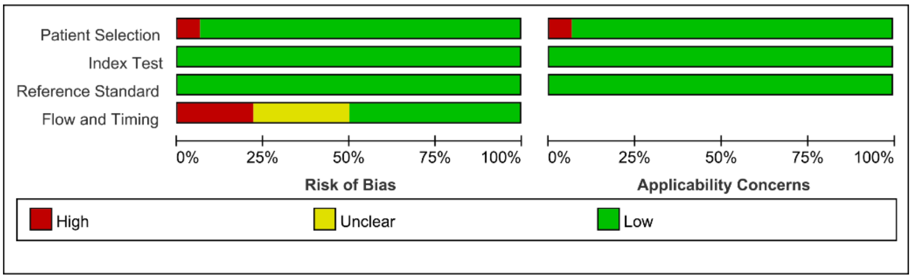

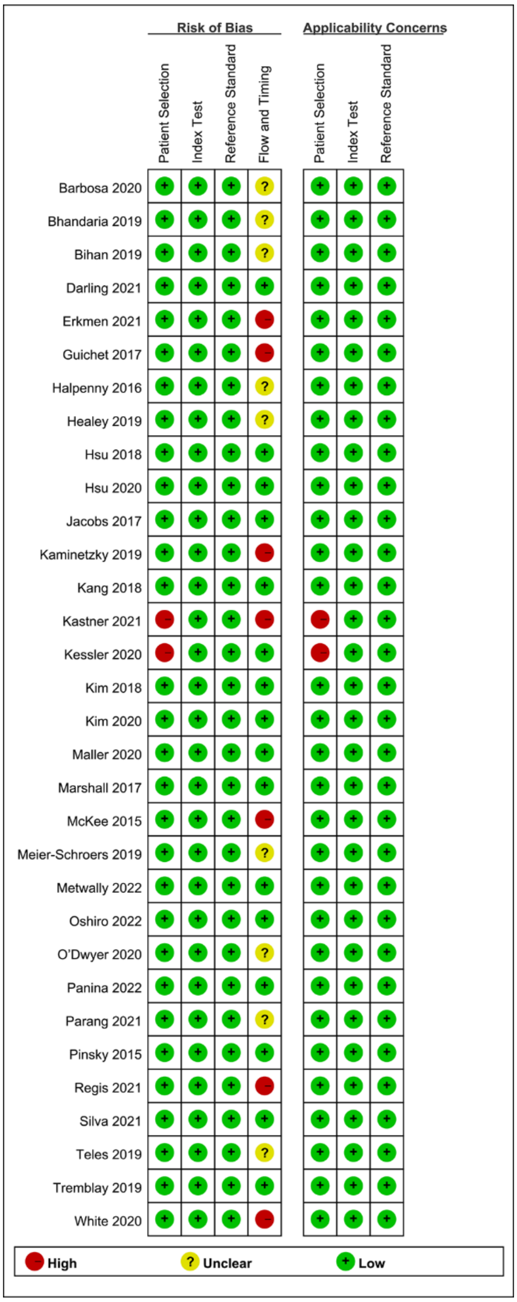

Figure S1. The quality assessment results of 31 included studies by the Quality Assessment of Diagnostic Accuracy Studies (QUADAS-2) tool: (a) Methodological quality graph. (b) Summary of rating scores regarding the risk of bias and applicability concerns.

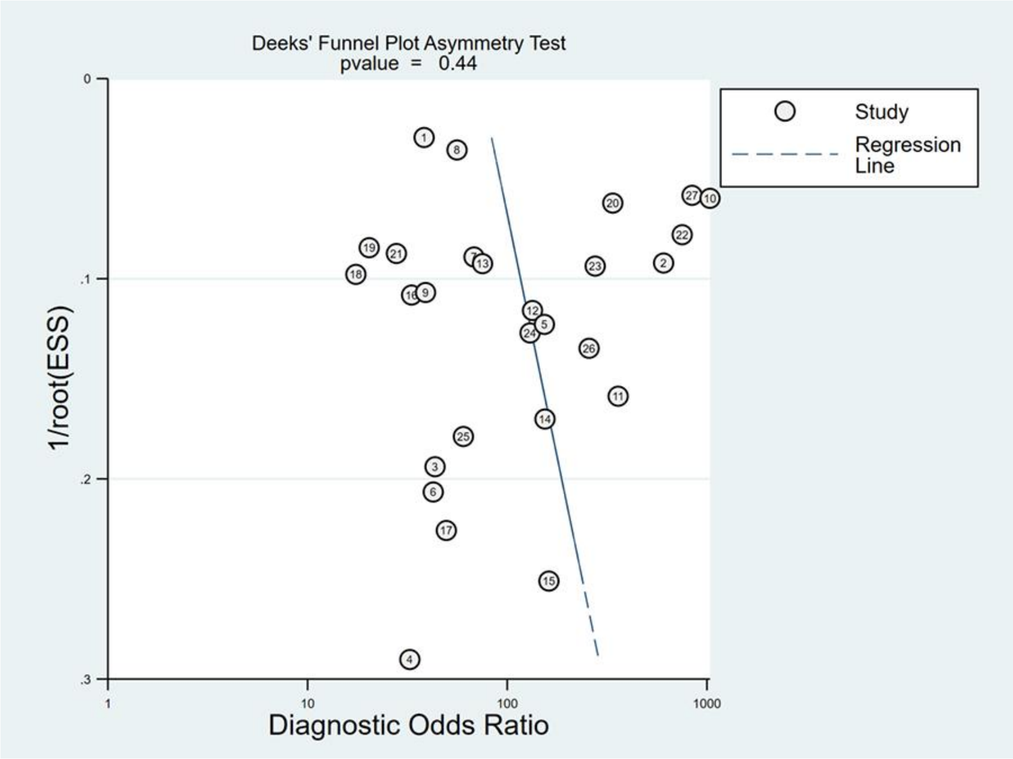

Figure S2. Deek's funnel plots for studies that assessed the Lung-RADS version 1.0.
